# Pigmentary complications after non-medical male circumcision

**DOI:** 10.1186/s12894-022-00999-5

**Published:** 2022-04-04

**Authors:** Radwa Tirana, Doa Othman, Dalia Gad, Menan Elsadek, Mohamed A. Baky Fahmy

**Affiliations:** Al-Azhar Faculty of Medicine for Girls, 21 Ibrahium Anes St, Al Nozha, Cairo, 11834 Egypt

**Keywords:** Male circumcision, Complications, Melanoma, Lentigo, Flickers, Kissing melanoma

## Abstract

**Background:**

A wide spectrum of complications are reported after male circumcision (MC), the non-aesthetic complications are well known, but the pigmentary complications scale are not reported precisely.

**Methods:**

This is a prospective cohort study of 550 circumcised boys; aged from 6 months to 14 years (62% aged 5 years) who were examined and appropriately investigated for the incidence of pigmentary complications after circumcision. Most diagnoses were clinically, but dermoscopy was done for 17 case and a skin biopsy for 14 cases. Patients with personal or family history of vitiligo, or congenital nevi were excluded. Available hospital records details and parents' statements were revised. The main outcome measures are the incidence of different pigmentary complications and circumcision details; data were analyzed by a non-parametric tests including the Mann–Whitney U test.

**Results:**

69 cases had 72 confirmed pigmentary complications discovered at 2–36 months after commencement of circumcision (mean 18). 48 cases had pigmentary complications directly related to MC, 11 cases were probably related and 10 unrelated to MC. The most common lesion is the circular hyperpigmented scar (29 cases); liner hyperpigmented scar in 13, spotted exogenous melanosis in 18 cases, melanocytic nevi (7), hypopigmentation diagnosed in 3 cases, but kissing nevus is the rarest finding (2). Topical corticosteroid was tried in 15 cases, surgical excision of pigmented scar were done for 19 cases, local laser used for 4 resistant cases and reassurance with follow up for the rest.

**Conclusion:**

Pigmentary complications after male circumcision are not rare and its management is challenging.

## Introduction

Male circumcision (MC) is usually practiced for many reasons, such as social, religious, or cultural, but rarely for medical reasons. This procedure is considered one of the oldest and most common surgical procedures practiced globally, recently, the MC rate across several countries has declined. This declining rate may reflect the changes in demographic patterns and parental beliefs raised by studies in psychology and ontogeny [1]. There is a wide spectrum of post MC complications. Abnormal penile pigmentations frequently affect men’s physical and psychological wellbeing; they develop slowly and can be very extensive with late effects on sexual function and self-esteeming. The incidence and prevalence of post MC pigmentary complications are unknown, and there is a clear evidence gap in the literature, with only a few case reports describing these pigmentary complications after MC [2]. Mostly, genital hyperpigmentation is constitutive and racially variable. However, facultative pigmentation induced by sun rays is rarely relevant in the genital area, but postinflammatory hyperpigmentation is common. Linear hyperpigmentation along the median raphe of the ventral penile shaft is usually seen as normal. Melanocytic lesions are considered the cause of genital pigmentation, but postinflammatory hyperpigmentation that occurs after inflammatory dermatosis, such as lichen planus, is much more commonly encountered [3]. Genital melanosis is characterized by increased pigmentation of basal keratinocytes and melanocytes, but there is no increase in melanocyte number. If melanocytes are present in increased numbers, the term ‘genital lentiginosis’ is more appropriate, genital freckles are usually seen as one or more brown punctate macules on the skin of the genitalia [4]. Minor cases of abnormal genital hyperpigmentation may only need patient or his parent's assurance and follow-up. Local corticosteroids may improve some cases; others may be ablated by laser therapy, but a disfigured pigmented scar deserves excision, especially if it is associated with bending or skin bridging over the glans [5].

## Patients and methods

A systematic genital examination was performed for all children and adolescents attending the outpatient clinic for genitourinary diseases or MC follow-up from June 2016 to March 2020. The examination commenced sequentially for the circumcision scar, coronal sulcus, frenular remnants, penile shaft, glans, and urinary meatus.

A systematic general examination was performed for the detection of other pigmentary or vascular lesions, especially in the buccal mucosa. Examination of the scrotum and scrotal contents and palpation of inguinal lymph nodes were also conducted. Onset and progression of the pigmentary lesions, previous use of drugs, or other remedies were reported. A detailed history of the circumcision was obtained concerning the timing of MC, method, type of anesthesia, bandage (type and for how long applied), previous use of drugs, and progression of post MC complications; type and management were reported. Dermoscopy was performed for 17 cases, and a punch or excisional biopsy with histological examination was performed for doubtful and suspicious cases (14/69). Minor cases of linear and/or circular scar hyperpigmentation are only followed up for 6 months for any progression or regression. Topical corticosteroids tried for cases with anxious and worried parents, surgical excision was performed for cases associated with other MC complications, such as skin bridges or cicatricial phimosis, laser technique was used effectively in 4 cases. Data were analysed by nonparametric tests, including the Mann–Whitney U test. A *P* value less than 0.05 was considered significant.

## Results

The total number of cases examined was 550 males aged 6 months to 14 years (mean 5 years), 69 cases (12.5%) had 72 confirmed pigmentary complications (three of them had combined lesions), and all PCs were discovered at 2–36 months after the commencement of circumcision (mean 18 months). Parents were aware of the existence of PC in only 28 cases; 12 patients were mindful about the lesion himself, but 29 cases were detected accidentally during the study.

**Fig. 1:**
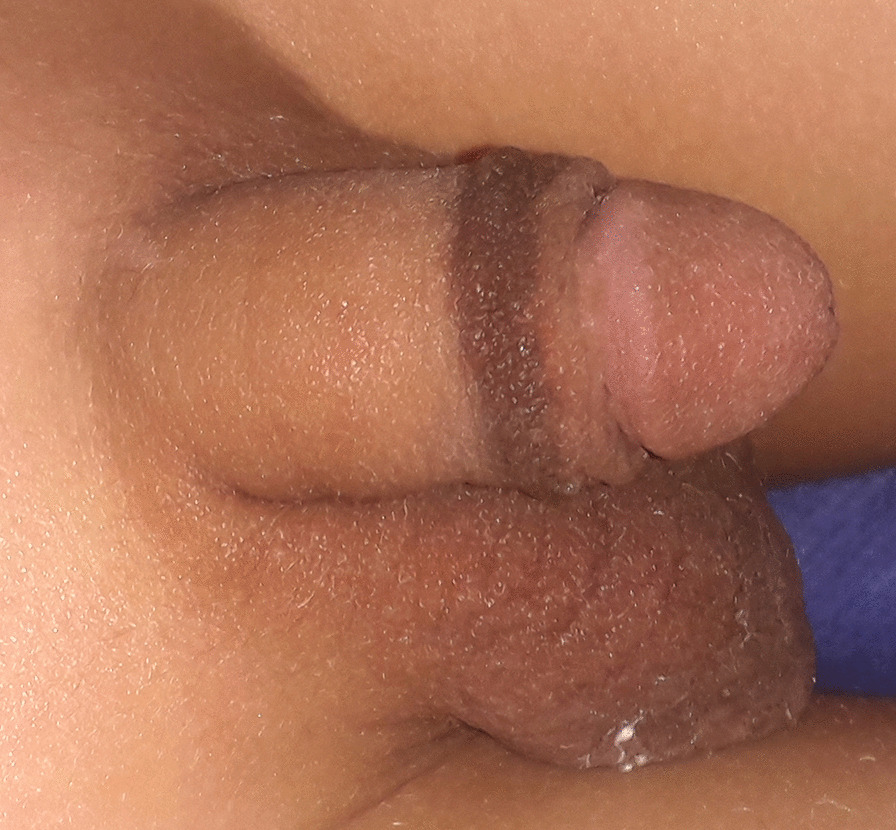
Circular hyperpigmented scar proximal to the coronal sulcus in a high loose circumcision scar

The most common lesion detected was a circular hyperpigmented scar proximal to the coronal sulcus (n = 29); eighteen of them had a highly loose circumcision scar, (Fig. 1) 4 had a redundant excess prepuce, two of them had a full picture of cicatricial phimosis, and 5 of those cases had variable degrees of skin bridges between the penile shaft and the glans.

**Fig. 2:**
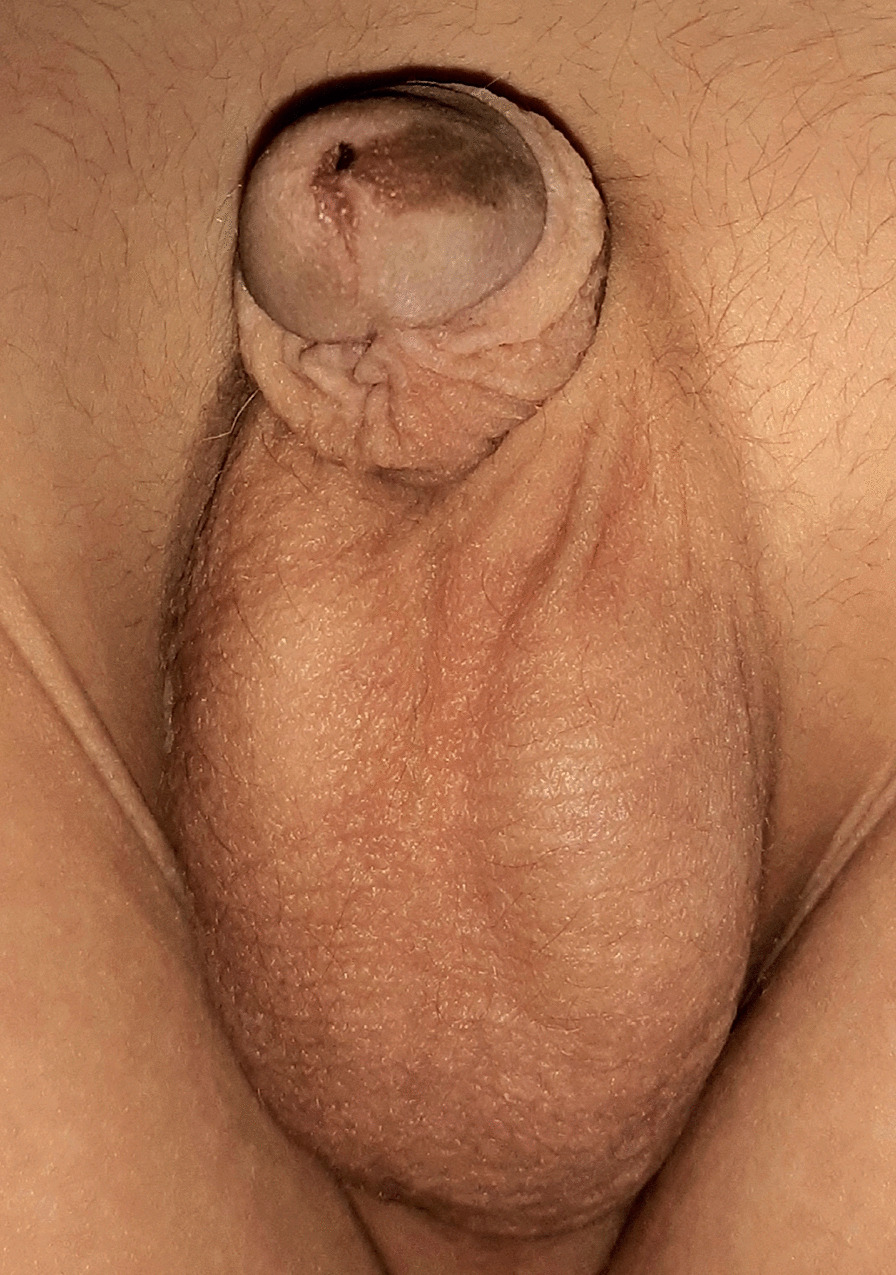
A linear hyperpigmented scar with a skin bridge over the glans

**Fig. 3:**
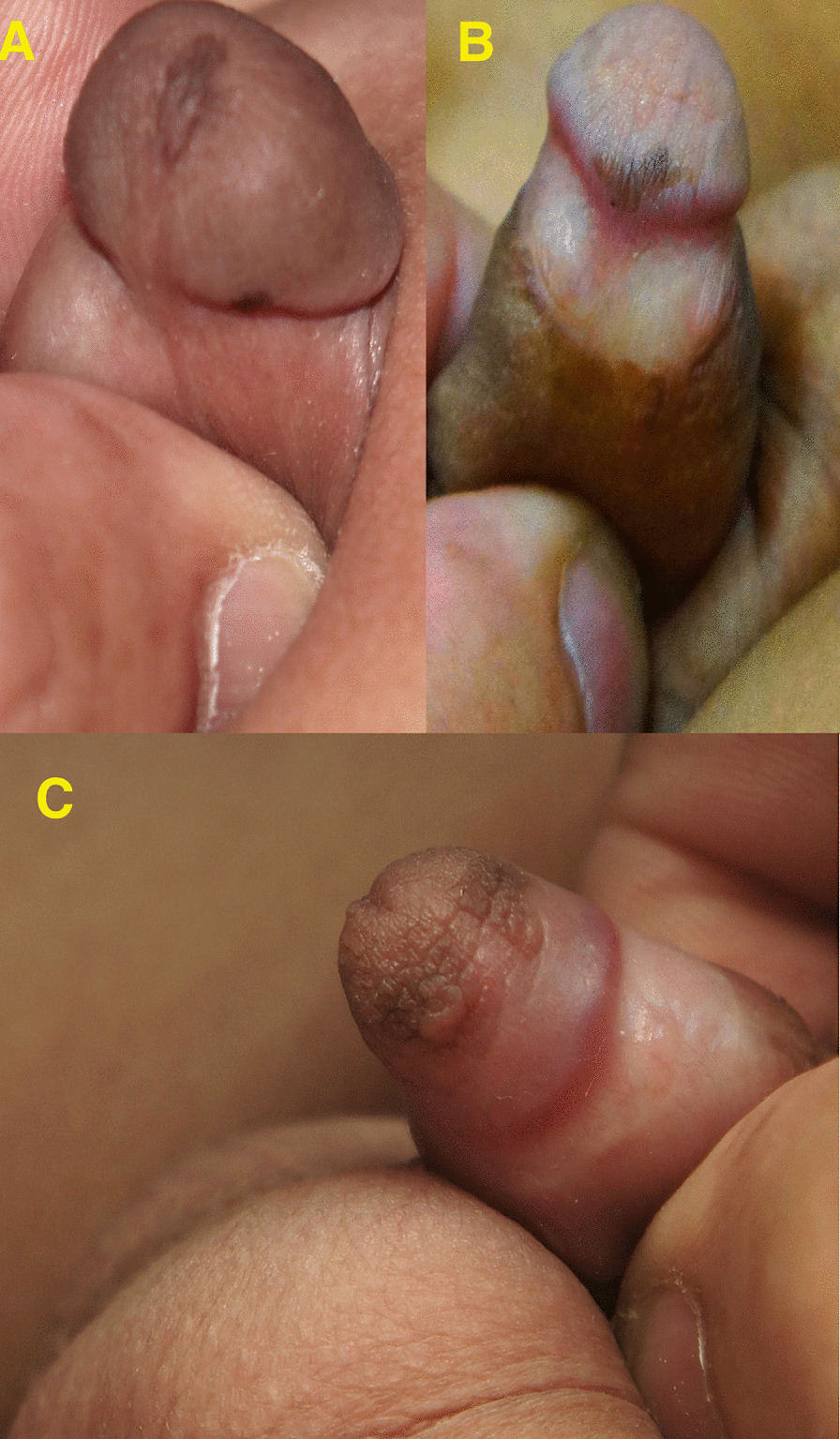
**A** Hyperpigmentation around the urinary meatus, which may be secondary to meatitis. **B** Proximal nevus at the dorsum of the glans near the coronal sulcus and **C** larger nevus over the glans

**Fig. 4:**
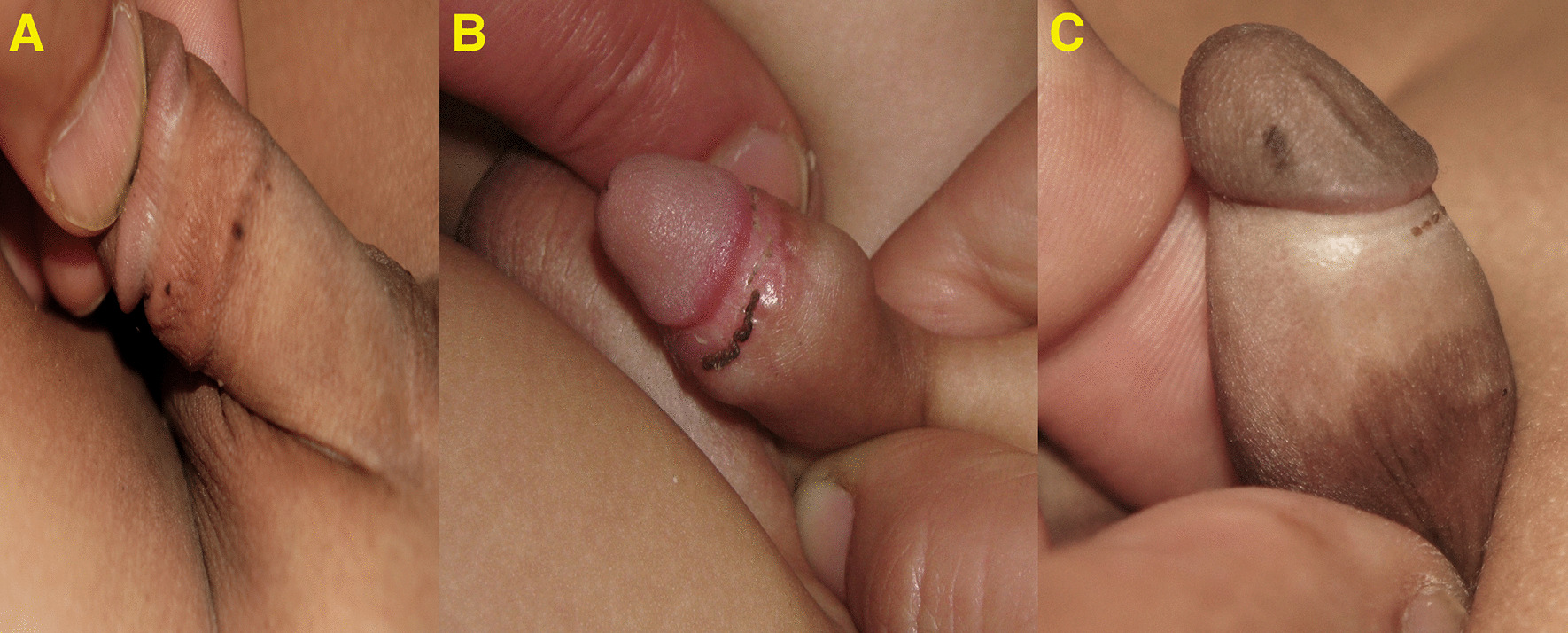
**A** Multiple small dark melanocytic nevi at the circumcision scar. **B** Hyperpigmented raised exogenous hyperpigmented scar at the preputial remnants. **C** Combined glandular melanosis, exogenous hyperpigmentation and circular preputial remnant hyperpigmentation

**Fig. 5:**
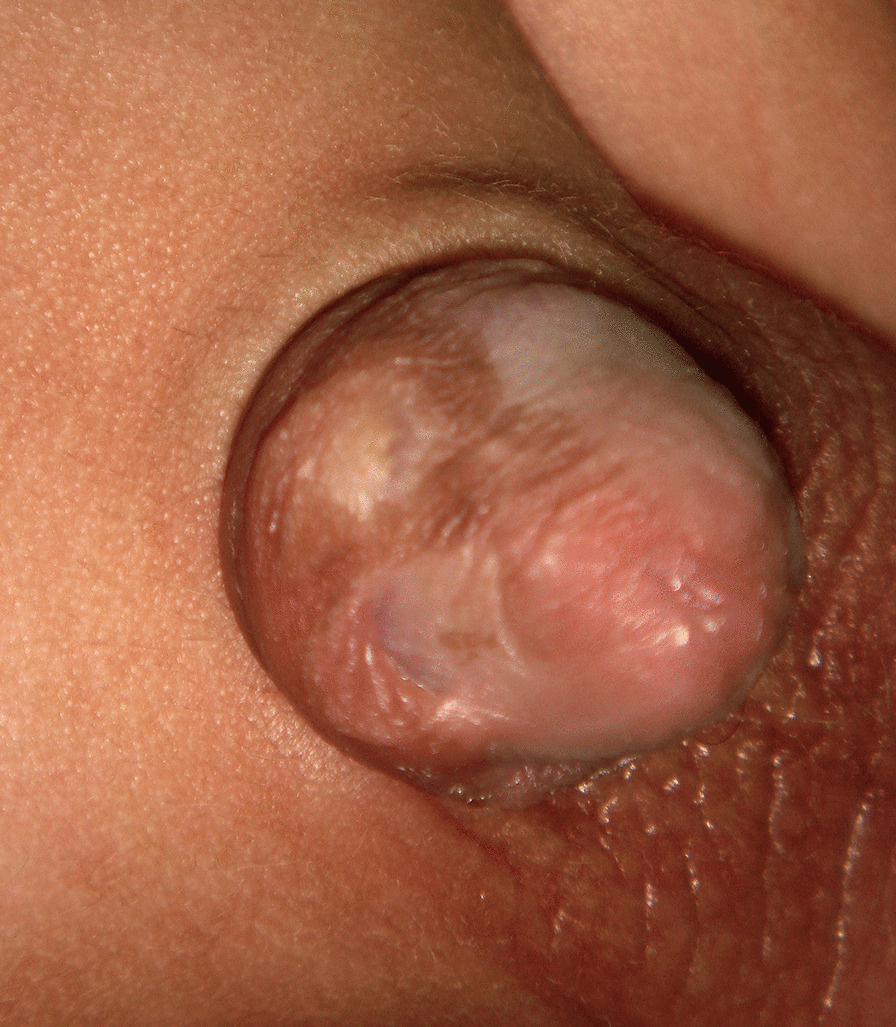
Post MC balanitis xerotica obliterans (BXO) presented with hyperpigmented glans scarring

**Fig. 6:**
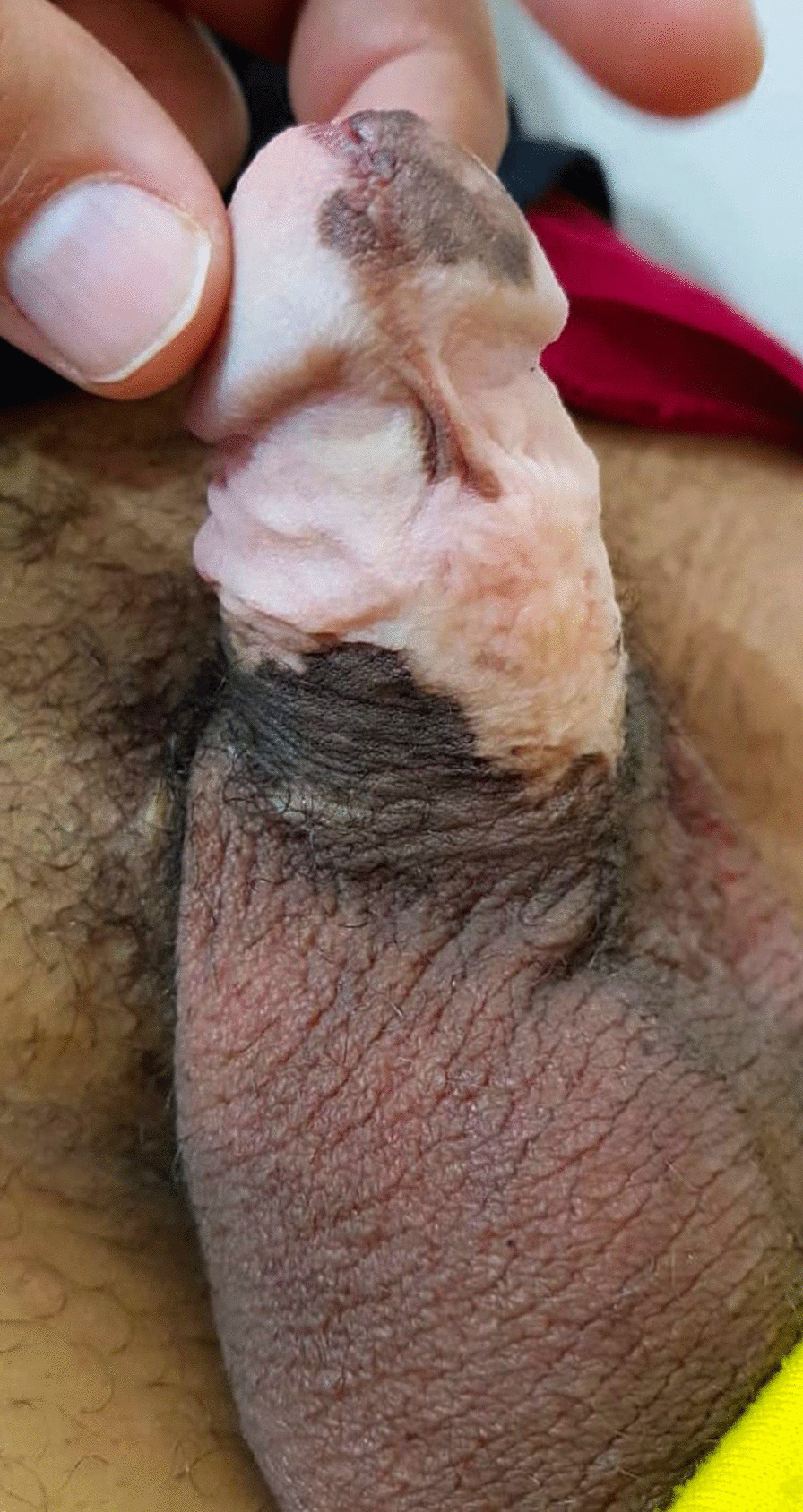
Hyperpigmented glans and proximal penile shaft with distinctive distal hypopigmentation

**Fig. 7:**
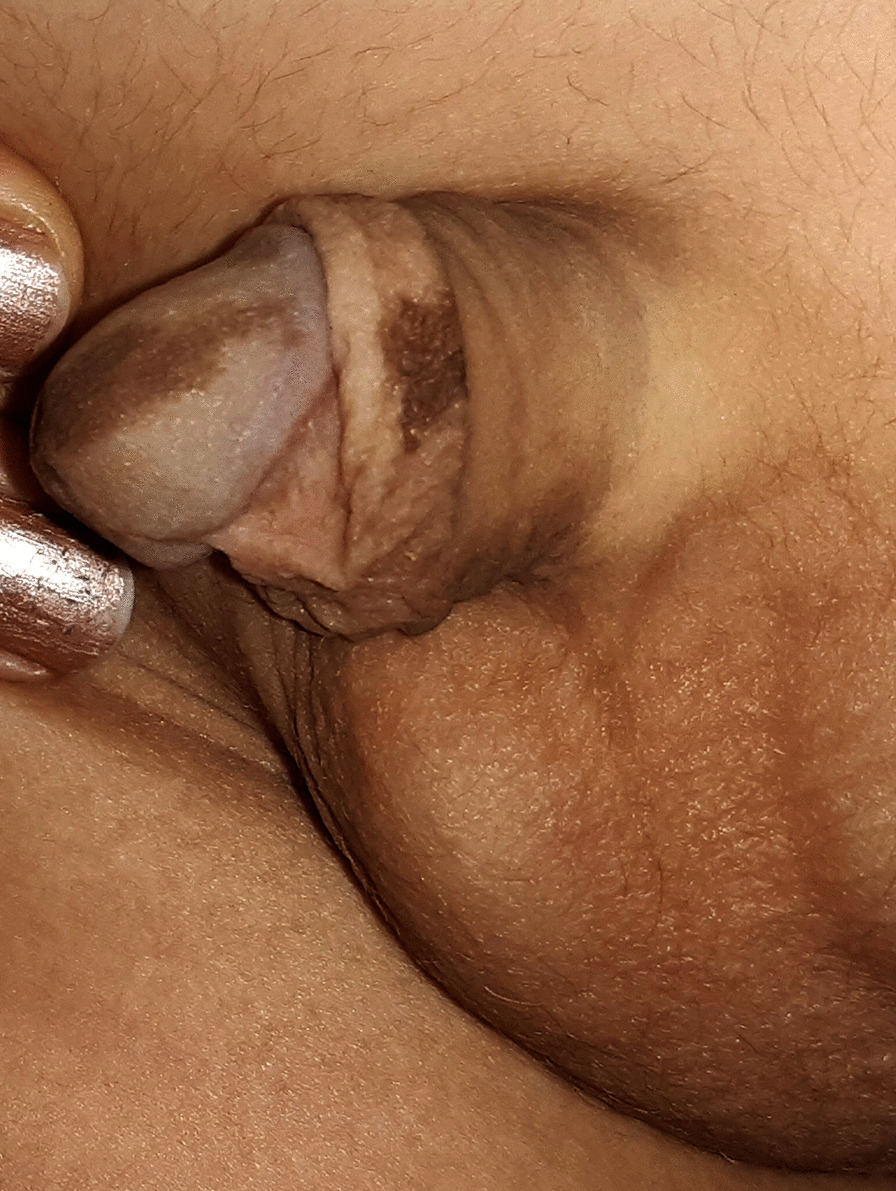
Kissing nevus at both the glans and the preputial remnants

Another 13 cases had linear pigmentation along the glans penis or the shaft of the penis (Fig. 2) or a combination of circular scar hyperpigmentation and a scared hyperpigmented ventral surface of the penile shaft; glanular hyperpigmentation was commonly seen around the meatus (Fig. 3a), different sizes of melanocytic nevi (glandular melanosis) on the glans or corona were diagnosed in 7 cases (Fig. 3b, c), and multiple small dark melanocytic nevi at the circumcision scar were diagnosed in 2 cases (Fig. 4a). Different forms of raised exogenous hyperpigmentations were diagnosed in 9 cases (Fig. 4b), and combined lesions were not rare (Fig. 4c). One case had balanitis xerotica obliterans (BXO), diagnosed histologically and presented with a hyperpigmented glanular scarring with skin bridges (Fig. 5). Different forms of hypopigmentation were diagnosed in 3 cases (Fig. 6), but kissing or divided nevus was the rarest complication (2 cases) (Fig. 7). History of post MC meatitis and different forms of balanitis subjectively confirmed in 23 cases, minor post MC bleeding in 6 cases, circumcision redo in 6. All cases reported that the child had a gauze bandage after the procedure; in 41 cases, the bandage was left for more than  one day, and the rest had an overnight bandage.

As much as the parents could not remember any abnormality or associated pathological conditions existed before circumcision, all of them denied any similar pigmentary lesion detected or reported prior to circumcision. Also all cases enrolled in the study have no history of systemic diseases which may be manifested by systemic or local pigmentation, and no history of specific drug eruption. Forty-five patients with PC were circumcised in the 1st month of life: 32 at the 1st week, 14 at 6 months, and 10 after 12 months of life. The circumcision methods in most cases were the bone cutting clamping guillotine method (42/69) under local anaesthetics in 28 cases ,but without anaesthesia in 14 cases, Gomco clamp method used in 18 cases, and plastibell used in 9 cases.

Betadine solution (povidone-iodine 10%) is an antiseptic nonsterile solution used by the mother for dressing the circumcision wound in 85% of the cases, which was accompanied by topical antibiotic ointment in 65% of the cases. In another 10 cases, the family was unacquainted about the method of post MC dressing. Topical corticosteroid was tried as management for minor PC with partial success in 15 cases, surgical excision of pigmented disfigured scar was done for 19 cases. Neodymium:yttrium–aluminum–garnet (Nd:YAG) laser technique was used effectively in 4 cases at or above the age of 8 years with extensive lentigo in 2 patients and nevi in another 2. Laser applied as a one session of Nd:YAG locally in the outpatient clinic, with a complete success in three of them, the parents of the fourth patient refused repetition of laser session. Only reassurance and follow-up were applied for the rest of the cases.

## Discussion

A circumcision procedure may be undertaken for modification of the genitals, to change the appearance of the penis and to appeal to more aesthetics. Nevertheless, it may leave a permanent change in the natal characteristics of the genitalia. Pigmentary complications may occur as a result of nonesthetic preputial cutting or untidy healing of the circumcision wound [6].

Pigmentary complications may appear after months or years of the procedure. Additionally, it may result in early family dissatisfaction and affect the psychological impact of a man’s contentment with his penis and may lead to loss of self-esteem [7].

Genital skin is relatively thin when compared with other sites, and the balanopreputial epithelium is incompletely keratinized, causing enhanced susceptibility to damage from irritants, resulting in several pigmentary complications, which may be transient or permanent, localized or segmental, or more rarely a combined lesion [8]. Cullen [9] proved that approximately 9% of males have at least one acquired melanocytic nevus during their lifetime in the genital area. Genitalia pigmentation is particularly influenced by sex hormone impregnation before birth, which leads to the normal dark color of the scrotum and median raphe. Other factors may partly explain hyperpigmentation, such as friction or chronic irritation, which may be manifested later after puberty.

Hyperpigmentation caused by postinflammatory conditions may coincide with an accelerated stimulation of genital skin melanocytes that leads to an increase in the melanin concentration in basal and suprabasal layers [10].

Postinflammatory hyperpigmentation may leave a scar that is darker than the surrounding unaffected skin. The mechanisms behind this are not fully understood but may involve activation of melanocytes by inflammatory mediators or reactive oxidative species released by damaged skin [11]. Macrophages then retain melanin in the dermis until the cells and melanin are degraded, enabling the pigment to persist within the dermis for some time postinjury. Increased and prolonged activation of melanogenesis within these lesions may rarely lead to uncontrolled proliferation and melanoma formation later on [12]. Certain factors predispose patients to a poor cosmetic appearance following MC; including insufficient hemostasis and failure to recognize anatomical diversities or abnormalities. Additionally, thick sutures with long absorption times, impertinent tissue handling, dressings that are too tight, and excessive resection of the prepuce are considered [13]. Generally, two-tonned penis and penile pigmentation variations are not rare among circumcised men, wherein the penis has two distinct colours. This two-tone penis may be considered ugly or of aesthetic concern for some men. In a survey completed by the National Organization of Restoring Men in the UK, 74% of the respondents were dissatisfied with the appearance of their circumcised penises, and 26% complained about the variation in skin color. In each case, the circumcision scar was clearly visible, and this scar was likely not of concern except after adulthood [14]. Freckles are commonly detected obviously at the glans penis after MC, but rare forms are difficult to diagnose and may mislead with other venereal diseases. Yazdanpanah et al. reported that genital warts were the most common disease diagnosed after MC among their cases, but inflammatory dermatoses such as psoriasis and balanitis were less common compared with other studies [15]. A lentigo is a small pigmented spot on the skin with a clearly defined edge surrounded by normal-appearing skin. It is a benign hyperplasia of melanocytes that is linear in its spread. This means that the hyperplasia of melanocytes is restricted to the cell layer directly above the basement membrane of the epidermis, where melanocytes normally reside. This is in contrast to the “nests” of multilayer melanocytes found in moles (melanocytic nevi). Stromal melanophages can occasionally be seen, especially in penile lentiginosis [16]. Lentigos are distinguished from freckles based on the proliferation of melanocytes, and freckles have a relatively normal number of melanocytes but an increased amount of melanin. Penile lentiginosis is characterized by the presence of multiple hyperpigmented small- to medium-size lesions with uniform or variegated pigmentation [17].

Diffuse hyperpigmentation resulting from chronic inflammation or postinflammatory conditions may present as multiple macules that give a ‘spotty’ appearance to the skin of the genitalia. A remarkable pigmentary loss in vitiligo occurs mainly in patients with dark complexions, and it may be overlooked in people with fair skin. Characteristically, it may appear as white patches on the glans penis, but spontaneous repigmentation has been known to occur. Hypopigmentation associated with postinflammatory conditions may be seen after any form of dermatophyte infection, genital ulcer, intertrigo, or chronic dermatitis [18]. In this series of patients, vitiligo was diagnosed in only 3 cases (Fig. 6).

Divided or kissing nevi, mostly located in the genitalia, are very rare and can only be seen on parts of the body where separation occurs during embryogenesis. Commonly, these nevi are believed to be benign pigmented tumors [19]. Divided naevus has been reported with one component located on the dorsal or dorsolateral side of the glans and the other on the distal penile shaft or inner face of the prepuce and separated by uninvolved skin across the coronal sulcus. It has been hypothesized that melanoblasts migrate to the lesion before embryological separation of the epithelial preputial placode [20]. It is difficult to incriminate circumcision as an etiology of such a type of nevus, and our finding of 2 cases of divided nevus may be a coincidental diagnosis. Penile melanosis had a particular clinical and microscopic appearance characterized by pigmented macules plus basal layer hyperpigmentation. It may be associated with or without melanocytic hyperplasia. Some patients with penile melanosis have local irritation, and injuries occur with circumcision or diabetes mellitus [21]. Post MC pigmentary complications can be differentiated from cases of fixed drug eruption, which may occur 1–2 weeks after initial exposure to oral medication. These lesions are mainly solitary, violaceous inflammatory plaques located on the penile shaft or glans. Furthermore, lesions may be associated with pain or pruritus and heal with time but may leave hyperpigmented patches. The circumcision procedure inherently limits the superficial vascular supply either through the mechanism of local vasospasm induced by the preputial excision itself or, alternatively, the pressure or tumescence of the homeostatic dressing wrapped around the penile shaft following MC, which may hinder the blood supply to some degree [22].

This cohort study was carried out on all circumcised boys attending the outpatient clinic during the period from June 2016 to March 2020. Common pigmentary complications diagnosed in this group of patients are circular and linear hyperpigmentation proximal to the coronal sulcus, which gives the penis the look of the two-toned penis (Fig. 1). In 48 cases, pigmentary complications were proven to be directly related to MC; linear, circular and freckle hyperpigmentation were the common lesions in this group, and hyperpigmentation around the urinary meatus was definitely acquired after circumcision. Eleven cases were probably related to circumcision; the common lesions of this group were hypopigmentation and multiple small dark melanocytic nevi. In 10 cases, the lesion was unrelated to MC, mainly in patients with dividing nevi. Overall, the most common lesion is the circular hyperpigmented scar (29 cases); liner hyperpigmented scar in 13, spotted exogenous melanosis in 18 cases, melanocytic nevi (7), hypopigmentation diagnosed in 3 cases, but kissing nevus is the rarest finding (2).

Topical corticosteroid was tried in 15 cases, surgical excision of pigmented scar was done for 19 cases, local laser used for 4 resistant cases and reassurance with follow up for the rest.

Penile melanosis is generally harmless and does not require treatment, but some people may choose to have cosmetic procedures to remove the spots. In hyperpigmented lesions, melanosomes are destroyed by the laser, preventing further melanogenesis and melanin transfer. Laser therapy can achieve good results but may require multiple treatments [23].

In this study, prolonged dressing for 2 or more days was significantly associated with hyperpigmentation (60% of the cases), and using 10% povidone-iodine was significantly associated with PC, especially circular and linear hyperpigmentation. Additionally, post MC infection in the form of balanitis, meatitis, or BXO was significantly associated with PC (24/69); *P* value less than 0.05.

This study intends to arouse awareness towards this hitherto of unrecognized complications, and better knowledge regarding it may assist in formulating an educated approach towards this complication to employ preventive measures to eliminate it or at least provide reassurance wherever it is possible.

## Study limitations

Lacking previous documented similar observations of these findings postulate that either inherent qualities of the penile skin or external factors inducing damage to the penile soft tissue are responsible for such complications. Also ethnical factor may play a role in our results; so another comparative multiethnic study may be more documentary. At the meantime the age of this sample of patients are confined to children; so a study of adults and elder population could be supportive to our results.

## Conclusion

Post MC penile hypo- or hyperpigmentation may appear after several conditions affecting the penis, such as balanitis and meatitis (33%), secondary to topical medications applied after MC or a tight bandage, which may have been the cause of the spotty pigmentation of the glans. Biopsy and histology are indicated when history is unclear or the causative agent is no longer present. Further follow up and appropriate therapy is mandatory for such cases.

## Data Availability

All raw data, material and other photos are available upon request from the corresponding author at (ymabfahmy@yahoo.com).

## Abbreviations

MC: Male circumcision
PC: Pigmentary complications
NdYAG: Neodymium:yttrium–aluminum–garnet
BXO: Balanitis xerotica obliterans
